# Quantitative assessment of the brain white matter myelination of patients with bipolar disorder using macromolecular proton fraction

**DOI:** 10.1192/j.eurpsy.2026.11215

**Published:** 2026-07-31

**Authors:** T. Amstislavskaya, S. Gusakova, O. Borodin, E. Epimahova, L. Smirnova, S. Ivanova

**Affiliations:** 1 Scientific Research Institute of Neurosciences and Medicine, Novosibirsk; 2 Siberian State Medical Universit; 3 State Regional Autonomous Budget Health Care Institution “Tomsk Regional Oncology Center”; 4 Laboratory of Molecular Genetics and Biochemistry, Mental Health Research Institute Tomsk National Research Medical Center Russian Academy of Sciences, Tomsk, Russian Federation

## Abstract

**Introduction:**

Introduction. In 2021, the global population of people with bipolar disorder (BD) was estimated at 37 million (or 0.5% of the global population), including approximately 34 million adults. Molecular proton fraction (MPF) has been recognized as a quantitative MRI parameter closely related to the myelin content of neural tissues and has already been used for non-invasive measurement of myelin deficiency in white matter (WM) and gray matter of patients with schizophrenia (Smirnova et al., Transl. Psychiatry, 2021).

**Objectives:**

To study changes in white matter myelination in BD.

**Methods:**

The main group consisted of patients (n = 18, including 13 women (72%), 5 men (28%), average age 27.5 ± 10.2), who were diagnosed with bipolar affective disorder (BD) according to ICD-10 criteria. All patients, as well as 14 patients in the control group, matched for sex and age, underwent magnetic resonance imaging of the brain using a 1.5T MR scanner (Ingenia Evolution, Philips, Netherlands) according to the standard protocol and the rapid MPF mapping protocol. Further segmentation of the brain masks was performed using FSL software (FMRIB Software Library, v. 6.0). Group differences were evaluated using the Mann–Whitney U test (p < 0.05) and the Pearson`s correlation test in Statistica 10.0.

**Results:**

Assessing the average values of white matter MPF of the brain obtained by automatic segmentation, there was a significant increase in white matter MPF revealed in patients with bipolar disorder compared to the control group (p=0.02) (Fig. 1). There was no significant direct correlation between the MPF value and the duration of the disease (r=-0.08, p-value = 0.7631), as well as between the MPF value and the age of disease onset (r=0.2, p-value = 0.42).

**Image 1:**
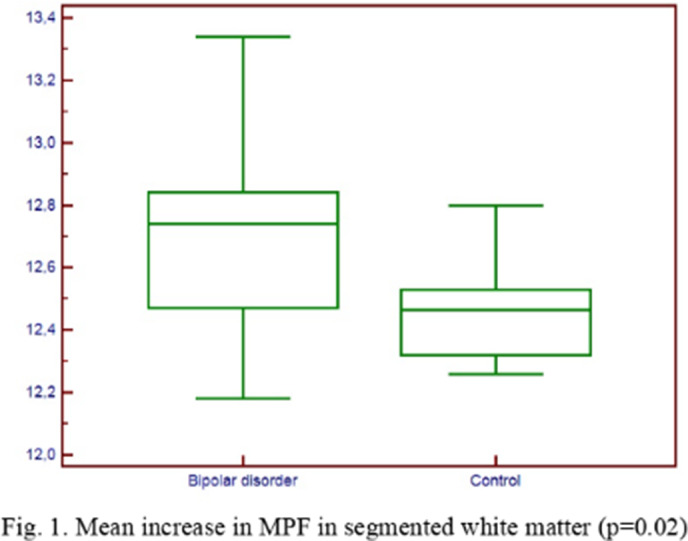
Image 1: Long description.

**Conclusions:**

Conclusions. The presence of a reliably significant increase in MPF in patients with bipolar disorder compared to the control group indicates a diffuse process of compaction of white matter myelin in this pathology. However, to reliably confirm the obtained results and identify indirect correlations, further larger-scale and standardized studies are required.

The study was supported by the Russian Science Foundation grant No. 23-75-00023.

**Disclosure of Interest:**

None Declared

## Long descriptions

### Image 1: Long description

The y-axis represents numerical values ranging from 12.0 to 13.4 in increments of 0.2. The x-axis identifies two categories: Bipolar disorder on the left and Control on the right.

* The Bipolar disorder box plot shows a median line at approximately 12.75. The interquartile range box extends from roughly 12.48 to 12.85. The lower whisker reaches down to 12.18 and the upper whisker extends significantly higher to 13.35.

* The Control box plot shows a lower distribution with a median line at approximately 12.45. The interquartile range box extends from roughly 12.32 to 12.53. The lower whisker reaches 12.25 and the upper whisker reaches 12.80.

The Bipolar disorder group exhibits both a higher mean and a wider range of M P F values compared to the Control group.

Navigate back to Image 1:.

